# Distinct alterations of retinal structure between thalamic and extra‐thalamic subcortical infarction patients: A cross‐sectional and longitudinal study

**DOI:** 10.1111/cns.14543

**Published:** 2023-11-29

**Authors:** Ruosu Pan, Chen Ye, Zhimeng Zhang, William Robert Kwapong, Ruilin Wang, Kun Lu, Lanhua Liao, Yuying Yan, Tang Yang, Le Cao, Shuai Jiang, Xuening Zhang, Junfeng Liu, Wendan Tao, Bo Wu

**Affiliations:** ^1^ Department of Neurology West China Hospital, Sichuan University Chengdu China; ^2^ Center of Cerebrovascular Diseases West China Hospital, Sichuan University Chengdu China; ^3^ West China School of Medicine Sichuan University Chengdu China; ^4^ Department of Ophthalmology West China Hospital, Sichuan University Chengdu China

**Keywords:** optical coherence tomography, retina, retrograde degeneration, single subcortical infarction, strategic infarction lesion location

## Abstract

**Aims:**

Cerebrovascular lesions in the primary visual cortex, the lateral geniculate nucleus, and the optic tract have been associated with retinal neurodegeneration via the retrograde degeneration (RD) mechanism. We aimed to use optical coherence tomography (OCT) to assess the effects of the strategic single subcortical infarction (SSI) location on retinal neurodegeneration and its longitudinal impacts.

**Methods:**

Patients with SSI were enrolled and stratified by lesion location on cerebral MRI into the thalamic infarction group and extra‐thalamic infarction group. Healthy controls from the native communities were also recruited. Retinal nerve fiber layer (RNFL) and ganglion cell‐inner plexiform layer (GCIPL) were quantified using OCT. Generalized estimating equation (GEE) models were used for cross‐sectional analyses and linear mixed models for longitudinal analyses. *P* < 0.05 was considered statistically significant.

**Results:**

We included a total of 283 eyes from 149 SSI patients. Of these, 115 eyes of 60 patients with follow‐up were included in the longitudinal analyses. Cross‐sectionally, thalamic‐infarction patients had reduced retinal thickness compared with extra‐thalamic infarction patients after adjustment for age, gender, disease duration, and vascular risk factors (*p* = 0.026 for RNFL, and *p* = 0.026 for GCIPL). Longitudinally, SSI patients showed greater retinal thinning compared with healthy controls over time (*p* = 0.040 for RNFL, and *p* < 0.001 for GCIPL), and thalamic infarction patients exhibited faster rates of GCIPL thinning in comparison with extra‐thalamic infarction patients (*p* < 0.001).

**Conclusion:**

Our study demonstrates a distinct effect of subcortical infarction lesion site on the retina both at the early stage of disease and at the 1‐year follow‐up time. These results present evidence of significant associations between strategic infarction locations and retinal neurodegeneration. It may provide novel insights for further research on RD in stroke patients and ultimately facilitate individualized recovery therapeutic strategy.

## Graphical abstract

We explored the cross‐sectional and longitudinal retinal structural thicknesses in SSI patients based on their location. We showed at the baseline that thalamic infarction patients had thinner RNFL and GCIPL thicknesses compared with extra‐thalamic stroke patients. Longitudinal analysis showed the thinning rate in GCIPL of the thalamic infarction group was significantly higher than that in the extra‐thalamic infarction group.

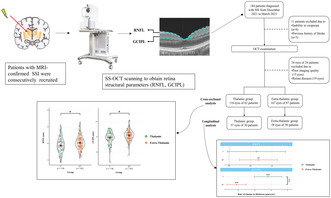

## 1 INTRODUCTION

Stroke, a debilitating cerebrovascular event, is one of the leading causes of mortality and long‐term disability worldwide.^1^ Secondary neurodegeneration induced by stroke is gradually being noticed and can be an unavoidable consequence.^2^ Since it is widely acknowledged that there is considerable homology in the retina and brain,^3^, ^4^ and retinal structural thicknesses may reflect cerebral structure,^5^, ^6^ a strong relationship between retina changes and stroke‐lesioned brain has been reported, that is, retinal secondary neurodegeneration after stroke.^7^, ^8^, ^9^ Previous studies have shown that quantitative changes in the retinal nerve fiber layer (RNFL),^10^, ^11^, ^12^, ^13^ ganglion cell complex (GCC)^14^ and ganglion cell‐inner plexiform layer (GCIPL),^15^, ^16^ measured by optical coherence tomography (OCT) tool, are associated with ischemic stroke. Importantly, these reports showed retinal structural thicknesses in ischemic stroke correlate with post‐stroke symptoms.^7^, ^8^, ^9^ These reports suggested that the OCT tool may have the potential to be used as a screening tool to monitor and detect secondary neurodegeneration in stroke.

Moreover, accumulating evidence has shown that the retina changes following stroke can exhibit considerable variability depending on the specific location of the lesion within the brain.^17^, ^18^ Previous studies found that patients with lesions situated along the visual pathway, that is, thalamus^19^, ^20^ or occipital lobe,^12^ may present more pronounced alterations in retina compared with other stroke locations. This phenomenon can be attributed to the unique mechanism of retrograde trans‐synaptic degeneration that occurs following damage to the visual pathway. To date, little is known about the specific retinal neurodegeneration changes in single subcortical infarction (SSI) patients. Understanding the impact of strategic stroke lesion locations on the retina structure changes is crucial for further comprehending the intricate relationship between the central nervous system and the retina.

As a common subcortical area of SSI and relaying center in visual pathway, thalamic infarction (TI) can lead to impaired visual field,^21^ visual acuity,^22^, ^23^ and oculomotor deficits.^24^, ^25^, ^26^ Importantly, neuro‐ophthalmic deficits following thalamic insults can significantly contribute to disease burden and cause severe disability, impacting various aspects of daily activities.^27^ Our previous research revealed that patients with thalamic infarction exhibited reduced RNFL and GCIPL thicknesses when compared with the control group^22^; besides, we showed thinning of the retinal structure correlated with visual acuity and microstructural optic tract changes in patients with thalamic infaction.^23^ Nonetheless, very little is known about the differences in retina structure between thalamic and extra‐thalamic SSI patients. Furthermore, it is widely acknowledged that the process of RD is time‐dependent,^12^, ^20^ which can have implications for stroke recovery and influence the variability of related therapies. Understanding the pathophysiology and clinical implications of stroke lesion location‐specific changes in the retina may provide valuable insights into the prognostic significance of retina examinations and RD mechanisms in stroke. Ultimately, it may facilitate personalized therapeutic approaches and enhance stroke patient care in the future.

Hence, in this study, we aimed to first, investigate and compare the distinct alterations in the structure of the retina between patients with thalamic infarctions and those with subcortical infarctions occurring in extra‐thalamic regions at baseline. Second, the relationship between lesion locations and the rates of retina layer changes was further explored in the longitudinal cohort.

## 2 MATERIALS AND METHODS

### 2.1 Study population

Patients with a radiologically confirmed single subcortical infarction (SSI), defined as our previous report,^6^ who visited the Department of Neurology, West China Hospital of Sichuan University, were cross‐sectionally and consecutively recruited between December 2020 and March 2023. The longitudinal analysis included patients who had at least one follow‐up visit at 1 year after the first examination. The SSI patients were classified, according to infarction location, into the thalamic infarction group and extra‐thalamic infarction group (infarction at basal ganglia, internal capsule, and/or corona radiata) by two experienced neurologists (C.Y. and Y.Y.). Age‐ and sex‐matched individuals without any neurological and ophthalmological disorders were recruited as healthy controls from the local communities through advertisement and were subjected to the same follow‐up visit protocol.

At the initial visit, demographic and clinical information were collected in a standardized format, including age, gender, and vascular risk factors (history of hypertension, diabetes, dyslipidemia, smoking, and drinking). Time since stroke (months, defined as the time in months between the date of index stroke and the OCT scan), National Institute of Health Stroke Scale (NIHSS) scores, and mRS (modified Ranking Scale scores) were also documented. Standardized neurological examinations were conducted by senior neurology residents (R.P. and C.Y.) under the guidance of one senior neurologist (B.W.). Participants enrolled in our study underwent extensive ophthalmologic examination including slit‐lamp biomicroscopy and ophthalmoscopy by an experienced ophthalmologist (R.W.) to exclude ophthalmic disorders which may affect the retinal structure. Inclusion criteria were as follows: (1) age ≥ 18, (2) had clinical diagnosis of first‐ever ischemic stroke due to SSI identified by MRI, (3) could cooperate with OCT retinal imaging, and (4) provided written informed consent. The exclusion criteria were as follows: (1) previous history of stroke or other neurological disorder, (3) diabetic retinopathy or other retinal diseases, (4) glaucoma, (5) poor OCT imaging qualities and (6) any contraindications for MRI examinations.

Written informed consent was obtained from each participant or their legal guardians, and this study was approved by the Ethics Committee of West China Hospital (No. 2020[922]).

### 2.2 Retinal imaging

Swept‐source optical coherence tomography (SS‐OCT, VG 200, SVision Imaging Limited, Luoyang, China) was used to image the structure of the retina by an experienced neuro‐ophthalmologist (W.R.K.). The specifications of the OCT tool have been well described in our previous report.^22^, ^28^

**FIGURE 1:**
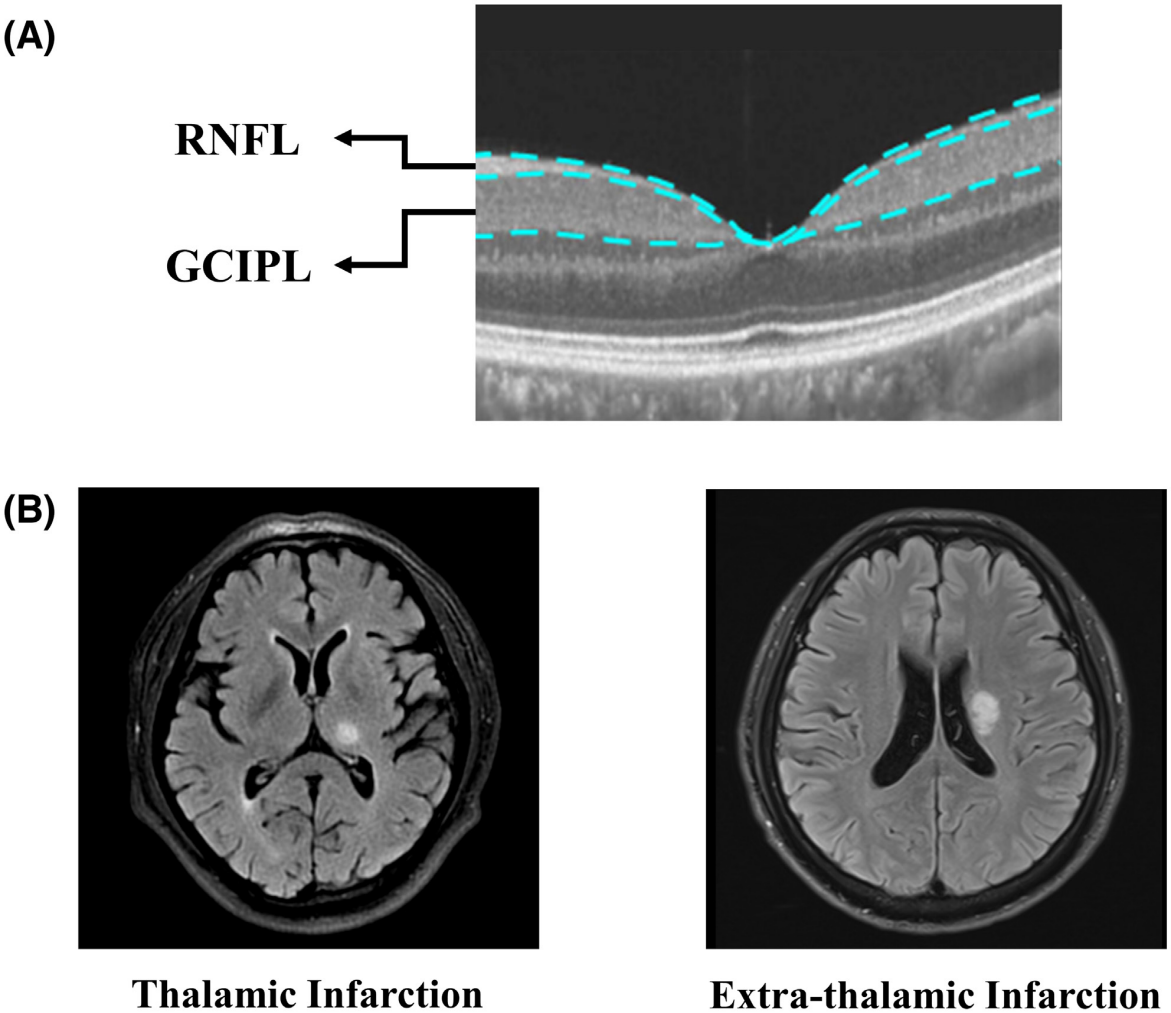
Representative images of OCT scans and MRI of patients. (A) The image shows the retina in a 3 × 3 mm^2^ area around the fovea. The OCT tool automatically segments the retina structure into RNFL and GCIPL. RNFL was defined as the thickness between the base of the inner limiting membrane (ILM) and the top border of the ganglion cell layer (GCL), and GCIPL was defined as the thickness from the base of the RNFL to the top border of the inner nuclear layer (INL). (B) The right image shows a fluid‐attenuated inversion recovery (FLAIR) MR image of a patient with thalamic infarction, and the left image shows an FLAIR image of a patient with extra‐thalamic infarction.

Structural OCT imaging of the macula was done with 18 radial scan lines focused on the fovea. Automatic segmentation of the retinal nerve fiber layer (RNFL) and ganglion cell‐inner plexiform layer (GCIPL) was done with a built‐in algorithm in the OCT tool. Specifically, RNFL was defined as the thickness between the base of the inner limiting membrane (ILM) to the top border of the ganglion cell layer (GCL), and GCIPL was defined as the thickness from the base of the RNFL to the top border of the inner nuclear layer (INL) as shown in Figure 1. Average thicknesses (measured in micrometers, μm) of the RNFL and GCIPL in a 3 × 3 mm area around the fovea were used in this study.

Images with ophthalmological disorders such as age macular degeneration, severe cataracts, diabetic retinopathy, and glaucoma were assessed by a neuro‐ophthalmologist and excluded. OCT data displayed in our study followed the OSCAR‐IB quality criteria^29^ and APOSTEL recommendation.^30^

### 2.3 Statistical analyses

Evaluation of normal distribution per variable was carried out via the Shapiro–Wilk test. Characteristics of the patients were described using mean (standard deviation, SD) or medians (interquartile range, IQR) for continuous variables, and numbers (percentages) for categorical variables where appropriate.

Baseline OCT measures were compared between the two groups with generalized estimating equation (GEE) model with age, gender, time since stroke and vascular risk factors (hypertension, diabetes, dyslipidemia, smoking, and drinking) controlled as covariates. The main outcomes were the absolute values of GCIPL and RNFL from baseline. For longitudinal analyses, the rates of change in retina measures were estimated using a linear mixed model with time since stroke, group as fixed effects, and subject/subject‐eye as random effects. The data are presented as positive reduction rates, which indicate a decrease in retina thickness over time. The analysis with the linear mixed model was adjusted for age, gender, and vascular risk factors (hypertension, diabetes, dyslipidemia, smoking, and drinking).^31^ In addition, we tested for differential rates of atrophy between thalamic group and nonthalamic group using interaction terms. The normality of residuals in the regression was checked by visual inspections of the normal quantile plot.

The statistical analyses were performed with R version 4.2.2 (http://www. R‐project.org) and *p* values of <0.05 were considered statistically significant. No correction for multiple comparisons was done since analyses were explorative.

## 3 RESULTS

### 3.1 Baseline characteristics

**FIGURE 2:**
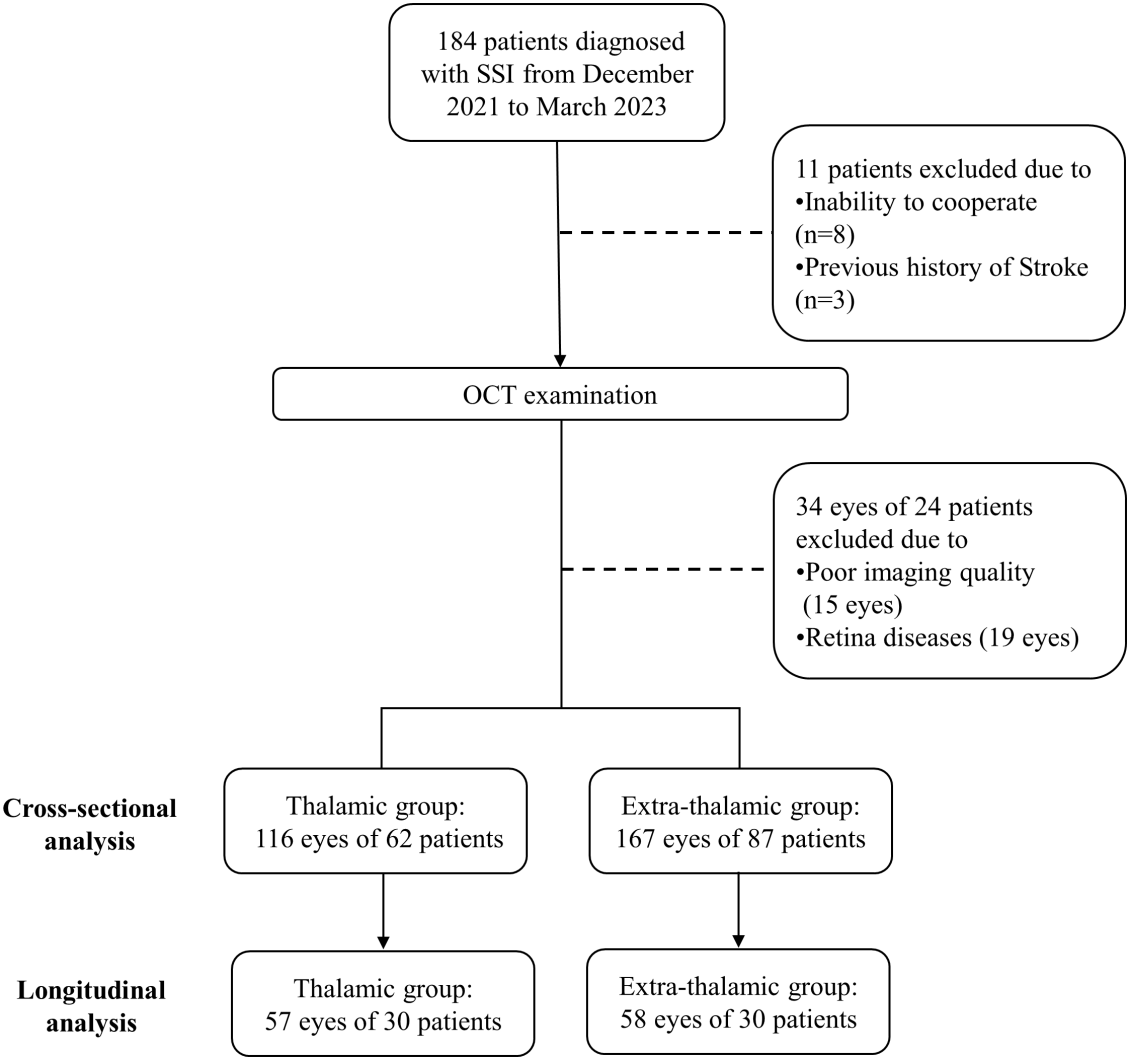
Flowchart of the patient inclusion. A total of 184 patients with SSI were included, of whom 3 patients with a prior stroke and 7 patients who failed to complete the OCT examination were excluded. Thirty‐four eyes were excluded due to poor imaging quality (15 eyes) or retina disease (19 eyes). Finally, a total of 283 eyes of 149 patients with single subcortical infractions (116 eyes of 62 thalamic infarction patients and 167 eyes of 87 extra‐thalamic infarction patients) were analyzed in this study. Of these, 57 eyes of 30 thalamic infraction patients and 58 eyes of 30 patients had follow‐up data. OCT, optical coherence tomography.

We enrolled 184 patients with SSI from December 2020 and March 2023 (Figure 2). Three patients with a prior stroke and seven patients who were unable to cooperate with OCT examination were excluded. Thirty‐four eyes were excluded due to poor imaging quality (15 eyes) or retina disease (19 eyes). A total of 283 eyes of 149 patients with single subcortical infraction (116 eyes of 62 thalamic infarction patients and 167 eyes of 87 extra‐thalamic infarction patients) were analyzed in this study. Of these, 57 eyes of 30 thalamic infraction patients and 58 eyes of 30 patients had follow‐up data. Patients' demographic and clinical data, stratified by infarct location, are displayed in Table 1. In cross‐sectional comparison, thalamic infarction patients showed lower NIHSS scores (1 [1, 2] vs. 3 [2–4], *p* < 0.001) and mRs (1 [1] vs. 2 [1–3], *p* < 0.001), and longer stroke duration since onset (1.42 [0.24–10.62] vs. 0.23 [0.17–0.45], *p* < 0.001) than extra‐thalamic infarction patients. No significant differences were found in age, sex, hypertension, diabetes mellitus, hyperlipidemia, smoking and drinking. In the longitudinal comparison, there were lower NIHSS scores (1 [1, 2] vs. 3 [1.25–5.75], *p* = 0.001) and mRs (1 [1] vs. 2 [1–3.75], *p* = 0.003), and lower rates of dyslipidemia (13.3% vs. 43.3%, *p* = 0.022) in thalamic infarction group, compared with extra‐thalamic infarction group. No significant difference was found in the follow‐up scanning time between the two groups (*p* = 0.496).

**TABLE 1:** Demographic and clinical information of patients.

	Thalamic infarction		Extra‐thalamic infarction		*p* ^a^	*p* ^b^
	Cross‐sectional	Longitudinal	Cross‐sectional	Longitudinal		
Patients, *n*	62	30	87	30	—	—
Eyes, overall, *n*	116	57	167	58	—	—
Ipsilateral, *n*	56	28	82	29	—	—
Contralateral, *n*	60	29	85	29	—	—
Lesion location, R, *n* (%)	33 (53.2)	15 (50.0)	38 (43.7)	15 (50.0)	0.325	1
Age, years, mean ± SD	58.58 ± 11.34	58.40 ± 12.51	55.22 ± 10.05	53.30 ± 10.13	0.058	0.088
Males, *n* (%)	45 (72.6)	23 (76.7)	73 (83.9)	25 (83.3)	0.14	0.747
Hypertension, *n* (%)	32 (52.5)	13 (43.3)	50 (57.5)	13 (43.3)	0.663	1
Diabetes, *n* (%)	18 (29.0)	6 (20.0)	27 (31.0)	13 (43.3)	0.935	0.096
Dyslipidemia, *n* (%)	16 (25.8)	4 (13.3)	29 (33.3)	13 (43.3)	0.421	**0.022**
Smoking, *n* (%)	30 (48.4)	14 (46.7)	49 (56.3)	14 (46.7)	0.429	1
Drinking, *n* (%)	27 (43.5)	14 (46.7)	34 (39.1)	10 (33.3)	0.706	0.429
Baseline NIHSS, median (IQR)	1 [1–2]	1 [1–2]	3 [2–4]	3 [1.25–5.75]	**<0.001**	**0.001**
Baseline mRS, median (IQR)	1 [1–1]	1 [1–1]	2 [1–3]	2 [1–3.75]	**<0.001**	**0.003**
Time since stroke at baseline scan, months, median (IQR)	1.42 [0.24–10.62]	1.32 [0.24–5.67]	0.23 [0.17–0.45]	0.22 [0.17–0.29]	**<0.001**	**0.003**
Time since stroke at last scan, months, median (IQR)	—	12.48 [7.11–21.62]	—	15.18 [13.40–18.98]		0.496

*Note*: Bold values indicate statistical significance (*p* < 0.05).

Abbreviations: mRS, modified Rankin Scale; NIHSS, National Institutes of Health Stroke Scale.

^a^ *p* values resulting from comparison between two groups in cross‐sectional data.

^b^ *p* values resulting from comparison between two groups in longitudinal data.

There were no significant differences between subcortical infarction patients and control participants with respect to age, gender, diabetes, drinking, and follow‐up time, whereas hypertension, dyslipidemia, and smoking were less frequent in the control group (Table S1).

### 3.2 Cross‐sectional comparison for retinal parameters in thalamic group versus extra‐thalamic group

**FIGURE 3:**
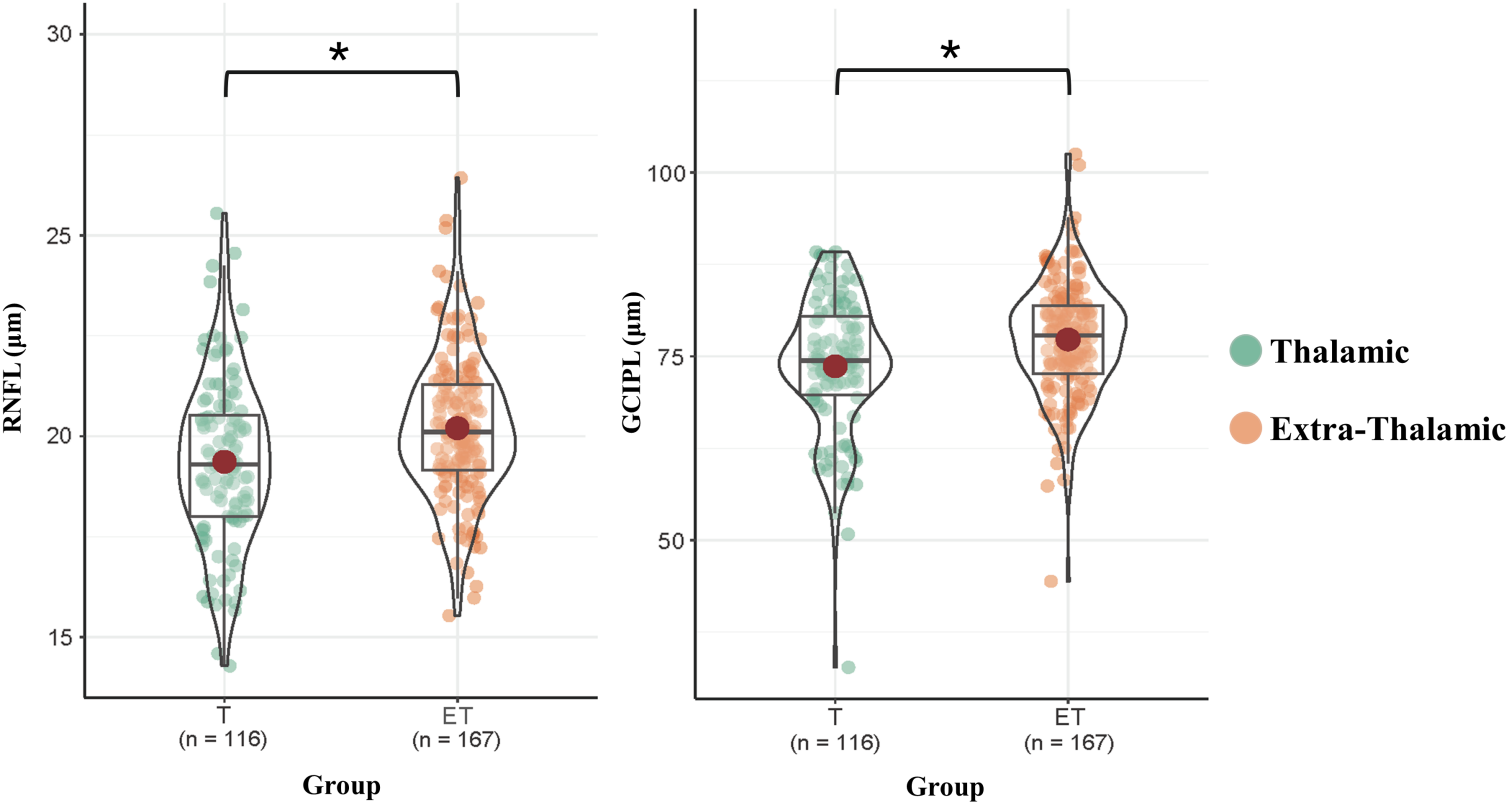
Cross‐sectional comparisons of retinal thickness at baseline. Thalamic infarction patients showed a thinner RNFL (*p* = 0.026) and GCIPL (*p* = 0.026) thickness compared with extra‐thalamic infarction patients after further adjustment for age, gender, time since stroke and vascular risk factors. ET, extra‐thalamic infarction group; GCIPL, ganglion cell‐inner plexiform layer; RNFL, retinal nerve fiber layer; T, thalamic infarction group. *p‐value < 0.05.

For cross‐sectional comparison, thalamic infarction patients showed a thinner RNFL *(p* = 0.026) and GCIPL (*p* = 0.026) thickness compared with extra‐thalamic infarction patients after further adjustment for age, gender, time since stroke and vascular risk factors (Table 2, Figure 3).

**TABLE 2:** Cross‐sectional comparisons of retinal thickness at baseline.

	Overall	Thalamic infarction	Extra‐thalamic infarction	*p*‐Value
Eyes, *n*	283	116	167	—
RNFL, μm mean ± SD	19.86 ± 1.96	19.36 ± 2.09	20.20 ± 1.79	**0.026**
GCIPL, μm, mean ± SD	75.80 ± 8.68	73.66 ± 9.34	77.29 ± 7.87	**0.026**

*Note*: Data were adjusted for age, gender, time since stroke and vascular risk factors (hypertension, diabetes, dyslipidemia, smoking, and drinking). Bold *p* values indicate statistical significance (*p* < 0.05).

Abbreviations: GCIPL, ganglion cell‐inner plexiform layer; RNFL, retinal nerve fiber layer.

### 3.3 Longitudinal retinal parameter changes in thalamic group versus extra‐thalamic group

Over time, the eyes of subcortical infarction patients showed reductions in both RNFL thickness (0.263 μm/year, 95% CI = 0.109–0.417 μm/year, *p* = 0.001) and GCIPL thickness (2.606 μm/year, 95% CI = 2.101–3.111 μm/year, *p* < 0.001) over the follow‐up time (Table S2). Healthy control only had thinning in GCIPL (0.973 μm/year, 95% CI = 0.623–1.323 μm/year, *p* < 0.001), while there was no significant change in RNFL of the healthy control group (*p* = 0.973) (Table S2). Subcortical infarction was significantly associated with a faster rate of RNFL (*p* for interaction = 0.04) and GCIPL (*p* for interaction < 0.001) thinning (Table S2).

**FIGURE 4:**
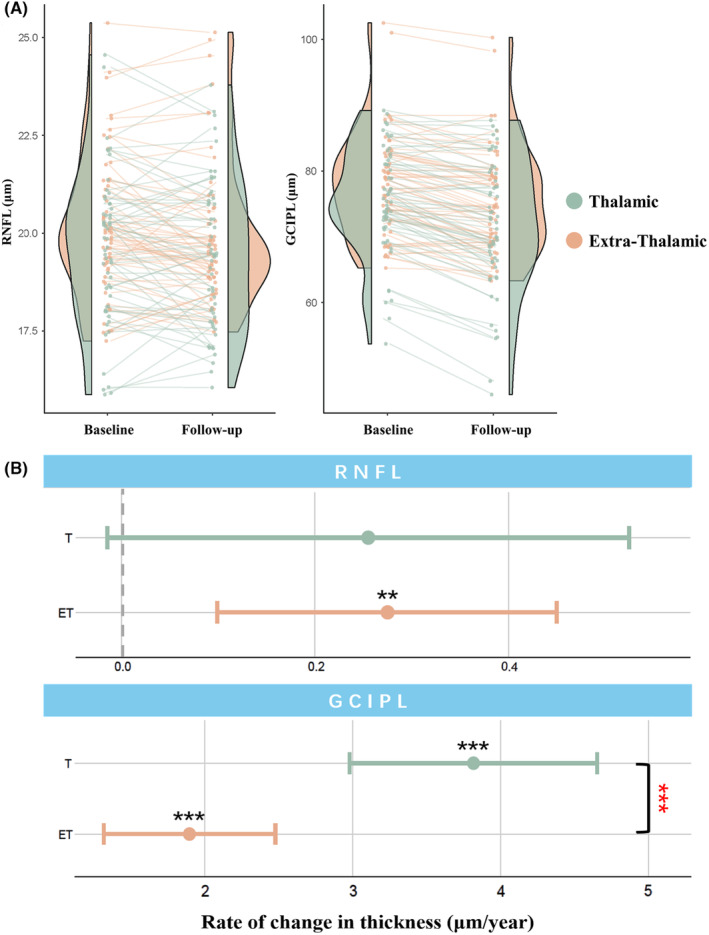
The atrophy rates of retinal thickness measures in the thalamic infarction group versus the extra‐thalamic infarction group. (A) The raincloud plot shows the change in retinal nerve fiber layer (RNFL) and ganglion cell‐inner plexiform layer (GCIPL) thickness in the thalamic infarction group (green) and in the extra‐thalamic infarction group (orange). (B) Thalamic infarction patients displayed significant thinning in the GCIPL thickness (*p* < 0.001). Extra‐thalamic infarction patients showed a significant decline in both GCIPL (*p* = 0.003) and RNFL (*p* < 0.001). The annualized atrophy rate in GCIPL of the thalamic group was significantly higher than that in the extra‐thalamic group (*p* for interaction <0.001). ET, extra‐thalamic infarction group; GCIPL, ganglion cell‐inner plexiform layerl; RNFL, retinal nerve fiber layer; T, thalamic infarction group. ** *p*‐values < 0.01, *** *p*‐values < 0.001.

Thalamic infarction patients displayed significant thinning in the GCIPL thickness (3.816 μm/year, 95% CI = 2.978 to 4.653 μm/year, *p* < 0.001). The change of RNFL in thalamic infarction patients showed a tendency to be thinner (0.255 μm/year, 95% CI = −0.015 to 0.525 μm/year, *p* = 0.067, Table 3, Figure 4). Extra‐thalamic infarction patients showed significant decline in both RNFL (0.275 μm/year, 95% CI = 0.099–0.45 μm/year, *p* = 0.003) and GCIPL (1.895 μm/year, 95% CI = 1.314–2.476 μm/year, *p* < 0.001) (Table 3; Figure 3).

**TABLE 3:** Longitudinal changes in retinal thickness.

	Thalamic infarction			Extra‐thalamic infarction			Thalamic vs. extra‐thalamic
	*n* = 57			*n* = 58			
	μm/year	95% CI	*p*	μm/year	95% CI	*p*	*p* for interaction
RNFL	0.255	−0.015 to 0.525	0.067	0.275	0.099 to 0.45	**0.003**	0.827
GCIPL	3.816	2.978 to 4.653	**<0.001**	1.895	1.314 to 2.476	**<0.001**	**<0.001**

Abbreviations: GCIPL, ganglion cell‐inner plexiform layer; RNFL, retinal nerve fiber layer.

*Note*: Data were adjusted for age, gender, time since stroke and vascular risk factors (hypertension, diabetes, dyslipidemia, smoking, and drinking). Bold values indicate statistical significance (*p* < 0.05).

Interestingly, the annualized atrophy rate in GCIPL of the thalamic infarction group was significantly higher than that in the extra‐thalamic infarction group (*p* for interaction <0.001) (Table 3, Figure 3).

## 4 DISCUSSION

Using the OCT tool, we aimed to explore the cross‐sectional and longitudinal retinal structural thicknesses in SSI patients based on their location. The atrophy rates of retina were also compared between different groups. We showed at the baseline that thalamic infarction patients had thinner RNFL and GCIPL thicknesses compared with extra‐thalamic stroke patients. Longitudinal analysis showed subcortical infarction patients, both thalamic and extra‐thalamic, had retinal neurodegeneration tendencies over time independent of normal aging. Importantly, we found a thinning rate in GCIPL of the thalamic infarction group was significantly higher than that in the extra‐thalamic infarction group.

Stroke is a heterogeneous disorder with respect to its location in the brain. It is suggested that structural changes in the retinal structural thicknesses may reflect neurodegeneration in the brain of individuals with stroke. Past studies showed individuals with ischemic stroke had thinner RNFL^10^, ^11^, ^12^, ^13^ and GCIPL^15^, ^16^ thicknesses compared with the control group. Baseline analysis in our study showed thalamic stroke patients had thinner RNFL and GCIPL thicknesses compared with extra‐thalamic stroke patients. The RNFL is mostly made up of retinal ganglion cell axons, whereas the GCIPL is made up of both the cell bodies and dendrites of the retinal ganglion cells; these structural thicknesses of the retina are used as markers of the structural integrity of retinal ganglion cells.^32^ Regarding the pathophysiology of our findings, it is plausible to suggest that infarct location plays an important role in retinal neurodegeneration. Infarction in the thalamus, a structure in the brain that has direct neural anatomical connections with optic tract and is involved in visual processing,^33^, ^34^, ^35^ may result in damage to connections within structures involved in vision, ultimately resulting in retinal sub‐layer neurodegeneration. Since the RNFL and GCIPL, are involved in visual processing, it is possible to suggest that secondary neurodegeneration in post‐thalamic stroke patients may be more severe than that in post‐extra‐thalamic stroke patients.

The retina is highly susceptible to ischemic stress because it is a metabolically demanding tissue. Previous reports showed GCIPL thinning is more sensitive to neurodegeneration in stroke patients compared with the RNFL thickness.^15^ Correspondingly, we showed GCIPL thinning was more severe in thalamic infarction patients compared with extra‐thalamic infarction patients, suggesting that GCIPL may be more informative and sensitive to post‐stroke secondary neurodegeneration, particularly in subjects with strategic lesion locations.

Interestingly, our longitudinal analysis showed the rate of neurodegeneration in the GCIPL was severe in thalamic stroke patients compared with extra‐thalamic stroke patients. Thinning of the RNFL and GCIPL is not only regarded as a ‘neurodegeneration phenomenon’ but also has been suggested as a useful indicator for monitoring the disease progression. Retinal sublayers decrease has been correlated with older age^36^ and incidence of certain disease.^37^ The deterioration of the GCIPL and preservation of the RNFL in thalamic stroke patients may indicate possible subtle deterioration of the dendritic complexity. The faster thinning of GCIPL in thalamic infarction patients might be noted to be an early onset and continuous neurodegeneration progression, even if there is an improvement in their neurological deficit (as measured by NIHSS). Due to its association with vision, we suggest that significant GCIPL thinning in thalamic infarction patients as shown through GCIPL thinning may be a sensitive biomarker of neurodegeneration. Further research with more comprehensive neurological evaluation, such as visual system tests or cognition, was needed to clarify and validate our hypothesis.

Our strength lies in the longitudinal analysis that enables us to capture retina changes of patients with different lesion location and overcome inter‐individual variation. There are some limitations in the present study. First, the follow‐up period was not the same in all participants which may yield variation of the changes. Further studies with similar and consistent follow‐up times are needed. Second, participants with ocular co‐morbidities and inability to undergo retinal assessment were excluded which might introduce selection bias. Third, although cerebral imaging was performed for all participants, cerebral imaging parameters were not assessed. In addition, simultaneously studying the changes in the retina and brain will further validate whether these structural changes could be developed as indicators for monitoring disease progression and therapeutic efficacy. Larger sample sizes with multiple OCT scan time points are warranted to confirm our findings and to characterize the trajectories of retina microstructure change after stroke.

In conclusion, we reported the longitudinal changes in retinal microstructures after subcortical infarction and the differential effect of lesion location1 on retinal neurodegeneration. These results provide novel insights for further research on RD in stroke patients and highlight the importance of subtyping strokes according to the lesion location. Further investigations are needed to elucidate the underlying mechanism and the clinical implications of these longitudinal retina microstructural alternations.

## AUTHOR CONTRIBUTIONS

Conceptualization and Design: **R.P., C.Y., W.R.K., and B.W.;** Data Collection: **R.P., Z.Z., L.C., W.R.K., K.L, H.L, Y.Y., T.Y., and S.J.;** Analysis and writing: **R.P;** Supervision: **R.W., J.F.L, and B.W.;** Writing: **R.P., C.Y., and W.R.K.;** Review and Editing: **C.Y., W.R.K., J.F.L, W.T., and B.W.** All authors approved the final manuscript.

## FUNDING INFORMATION

This study was supported by the Medical‐Engineering Integration Interdisciplinary Talent Training Fund Project of West China Hospital, Sichuan University and University of Electronic Science and Technology of China (HXDZ22011) and the Fundamental Research Funds for the Central Universities (ZYGX2022YGRH017); Sichuan Science and Technology Program (2019YJ0037); National Natural Science Foundation of China (82071320); and 1.3.5 Project for Clinical Research Incubation Project (2020HXFH012), West China Hospital, Sichuan University.

## CONFLICT OF INTEREST STATEMENT

The authors declared no potential conflicts of interest.

## Supporting information


Tables S1–S2

## Data Availability

All data generated or analyzed during this study are included in this published article. The detailed data sets used and/or analyzed during this study are available from the corresponding author upon reasonable request.
