# Supplementary material for: Distinct alterations of retinal structure between thalamic and extra‐thalamic subcortical infarction patients: A cross‐sectional and longitudinal study
Source: CNS Neurosci Ther. 2023 Nov 29;30(4):e14543. doi: 10.1111/cns.14543 (PMC11017429; doi:10.1111/cns.14543)
Supplement: Supplementary file 1 — Tables S1–S2 [file CNS-30-e14543-s001.docx]

## Supplementary Materials

## Supplementary Table 1. Demographic and clinical information of single subcortical infarction patients and healthy control

|  | Single Subcortical Infarction |  | Healthy Control | *P* |
| --- | --- | --- | --- | --- |
| Patients, n | 60 |  | 33 | - |
| Eyes, overall, n | 115 |  | 64 | - |
| Age, years, mean±SD | 55.78 (11.65) |  | 55.97 (13.69) | 0.945 |
| Males, n (%) | 49 (81.7) |  | 24 (72.7) | 0.459 |
| Hypertension, n (%) | 29 (48.3) |  | 8 (24.2) | **0.04** |
| Diabetes, n (%) | 22 (36.7) |  | 7 (21.2) | 0.192 |
| Dyslipidemia, n (%) | 17 (28.3) |  | 0 ( 0.0) | **0.002** |
| Smoking, n (%) | 28 (46.7) |  | 6 (18.2) | **0.012** |
| Drinking, n (%) | 24 (40.0) |  | 7 (21.2) | 0.108 |
| Follow-up duration,  months, median [IQR] | 12.97 [6.77-17.16] |  | 13.97 [11.20-18.67] | 0.143 |

mRS, modified Rankin Scale; NIHSS, National Institutes of Health Stroke Scale; SSI, single subcortical infarction.

Bold values indicate statistical significance (*P* < 0.05).

## Supplementary Table 2. Longitudinal changes in retinal thickness of single subcortical infarction patients and healthy control

|  | Single Subcortical Infarction | | | Healthy Control | | | SSI vs HC |
| --- | --- | --- | --- | --- | --- | --- | --- |
|  | n=115 | | | n=64 | | |  |
|  | μm/year | 95%CI | *P* | μm /year | 95%CI | *P* | *P* for interaction |
|  |  |  |  |  |  |  |  |
| RNFL | 0.263 | 0.109 to 0.417 | **0.001** | 0.007 | -0.179 to 0.193 | 0.942 | **0.040** |
| GCIPL | 2.606 | 2.101 to 3.111 | **<0.001** | 0.973 | 0.623 to 1.323 | **<0.001** | **<0.001** |

GCIPL, ganglion cell-inner plexiform layer; HC, healthy control; RNFL, retinal nerve fiber layer; SSI, single subcortical infarction.

Data were adjusted for age, gender, time since stroke and vascular risk factors (hypertension, diabetes, dyslipidemia, smoking and drinking).

Bold values indicate statistical significance (*P* < 0.05).
